# STAT3 Regulates Proliferation and Immunogenicity of the Ewing Family of Tumors *In Vitro*

**DOI:** 10.1155/2012/987239

**Published:** 2012-01-18

**Authors:** Sam Behjati, B. Piku Basu, Rebecca Wallace, Nelly Bier, Neil Sebire, Fyeza Hasan, John Anderson

**Affiliations:** ^1^Unit of Molecular Haematology and Cancer Biology, UCL Institute of Child Health, 30 Guilford Street, London WC1N 1EH, UK; ^2^Department of Histopathology, Great Ormond Street Hospital for Children NHS Trust, Great Ormond Street, London WC1N 3JH, UK

## Abstract

The Ewing sarcoma family of tumors (ESFT) represents an aggressive spectrum of malignant tumour types with common defining histological and cytogenetic features. To evaluate the functional activation of signal transducer and activator of transcription 3 (STAT3) in ESFT, we evaluated its activation in primary tissue sections and observed the functional consequences of its inhibition in ESFT cell lines. STAT3 was activated (tyrosine 705-phosphorylated) in 18 out of 31 primary tumours (58%), either diffusely (35%) or focally (23%). STAT3 was constitutively activated in 3 out of 3 ESFT cell lines tested, and its specific chemical inhibition resulted in complete loss of cell viability. STAT3 inhibition in ESFT cell lines was associated with several consistent changes in chemokine profile suggesting a role of STAT3 in ESFT in both cell survival and modification of the cellular immune environment. Together these data support the investigation of STAT3 inhibitors for the Ewing family of tumors.

## 1. Introduction

The Ewing sarcoma family of tumors (ESFT) is an aggressive malignancy of childhood comprising a spectrum of tumour types with common defining histological and cytogenetic features [1]. Overall mortality remains at around 40% despite the introduction of multimodality therapy, and current treatment protocols often cause significant long-term morbidity amongst survivors [2, 3]. The prognosis of ESFT is unlikely to change fundamentally, unless novel biological targets for treatment are identified. One interesting potential approach is to target the pathways that activate the transcription factor STAT3, which acts as a point of convergence of many different oncogenic signals [4, 5]. In particular, STAT3 has been shown to integrate pathways which regulate tumour growth and the immune microenvironment [6–8] and it is known to be deregulated in a range of adult cancer types [9–11]. However, the role of STAT3 in malignancies of childhood is poorly understood. Lai et al. demonstrated activated STAT3 (i.e., STAT3 phosphorylated at the tyrosine 705 residue) in approximately 50% of cases in a series of 49 ESFT tumours by immunohistochemistry [12]. In this study we sought to confirm their findings and investigate *in vitro* whether STAT3 integrates control of proliferation and the tumour microenvironment in ESFT tumors.

## 2. Materials and Methods

### 2.1. Tissue Arrays

Paraffin-embedded tumor samples were randomly selected from hospital histology archived material. Haematoxylin and eosin stained sections were examined to identify areas of viable tumour. Tissue cores were taken from these areas using a skin biopsy punch (diameter 4 mm) and reembedded to produce 3 tissue arrays, comprising a total of 31 tumour cores.

### 2.2. Immunohistochemical Analysis

Immunohistochemical analysis was performed on 4 *μ*m-thick sections, as previously described [13]. As primary antibody, the phosphorylated STAT3 (P-STAT3) monoclonal antibody clone 3E2 (Cell Signalling Technology, Boston, USA) was used at a dilution of 1 : 30. As positive control, tonsillar tissue was used. As negative control, the primary antibody was omitted. Analysis was performed by two authors, initially independently and then together to form a consensus opinion. The staining pattern was assessed qualitatively and also semiquantitatively using the following scoring system: 0, no reactivity; 1+, 1% to 10% positive cells; 2+, 10% to 50% positive cells; 3+, more than 50% positive cells.

### 2.3. Cell Culture and Formalin Fixation

The ESFT cell lines A673 and TC32 were kind donations of Professor Sue Burchill, University of Leeds, UK. The ESFT cell line SKNMC, the prostate cancer cell line PC3, and HeLa were obtained from ATCC. Cell lines were maintained in the following media by Gibco/Invitrogen (Pasley, UK): DMEM (cell lines A673, SKNMC, HeLa), RPMI (cell line TC32), or F12 (cell line PC3) media containing 10% foetal calf serum. For fixing of 20 ng/mL IL-6-treated HeLa cells (E-Bioscience), supernatant was aspirated after 10 minutes activation, and cell pellets were fixed in 10% formaldehyde for 10 minutes before undergoing processing as for routine histopathology specimens. Following processing, each sample was embedded in paraffin.

### 2.4. Western Blotting

For production of lysates the following experimental protocol was followed for ESFT and the PC3 cell lines. 500,000 cells were incubated in 2 mL media for 16 hours. Media was then removed and replaced by 1 mL of fresh media in the presence or absence of the pharmacological STAT3 inhibitor, S3i-201 (Calbiochem, Darmstadt, Germany), at a concentration of 100 *μ*mol/L. 24 hours later the cells were harvested and lysed using RIPA buffer (Pierce Biotechnology, Rockford, USA) containing protease inhibitor cocktail (Pierce Biotechnology, Rockford, USA) and sodium orthovanadate (Sigma-Aldrich, St. Louis, USA). Western blotting was then performed on equal amounts (in weight) of lysate as previously described [5], substituting the milk-containing blocking solution with 10% bovine serum albumin. The following primary antibodies were used with overnight incubations: P-STAT3 (clone 9131, 1 : 500 solution) and STAT3 (clone 79D7, 1 : 2000 solution), both from Cell Signalling Technology (Boston, USA), GADPH (1 : 2000 solution of clone G9545, Sigma-Aldrich, St. Louis, USA). As positive control for P-STAT3, lysates of IL-6-stimulated HeLa cells were used. As positive control for STAT3, lysates of HeLa cells cultured in the presence of 0% foetal calf serum were used.

### 2.5. Cell Viability Growth Assay

25,000 cells were plated in 96-well flat-bottom plates suspended in 100 *μ*L of media. 16 hours later, treatment was added. Each treatment condition was performed in triplicates. 24 hours later, cell viability was assessed, using 20 *μ*L of the CellTiter 96 Aqueous nonradioactive cell proliferation assay based on the tetrazolium compound [3-(4,5-dimethylthiazol-2-yl)-5-(3-carboxymethoxyphenyl)-2-(4-sulfophenyl)-2H-tetrazolium, inner salt, MTS (Promega, Madison, USA). Absorbance was measured using the 96-well plate BIO-RAD Model 680 microplate reader (Hercules, USA).

### 2.6. Chemokine Arrays and Array Analysis

Supernatants for chemokine array analysis were produced by following the experimental protocol for production of lysates, as outlined above. Before harvesting and lysing cells, spent media was removed and stored at −80°C, after centrifugation to remove debris. The supernatants were analysed at a later stage using the Ray Biotech (Norcross, USA) Human Chemokine Array 1 according to the manufacturer's instructions, using overnight incubations. The spots on the developed films were scanned with a BIO-RAD GS-800 Calibrated Densitometer (Hercules, USA) and quantified using ImageQuant software (GE Healthcare, Little Chalfont, UK). From each spot that represented a chemokine, the background (negative control spots) was subtracted. The chemokine spots were then normalised against positive control spots. The values that were obtained thus were density values expressed as percentage of positive control spots. Further analysis was then carried out using Microsoft Excel 2007 (Redmond, USA). Chemokines which had changed in control experiments (PC3 cell line) by more than 10% were excluded from further analysis, to exclude potential off-target effects. A change in chemokine level was arbitrarily defined as an increase or decrease by more than 10%, observed in arrays from two independent experiments.

## 3. Results

### 3.1. ESFT Are Characterised by a High Incidence of STAT3 Activation and Associated with Discrete Tissue Distribution Patterns

**Figure 1:**
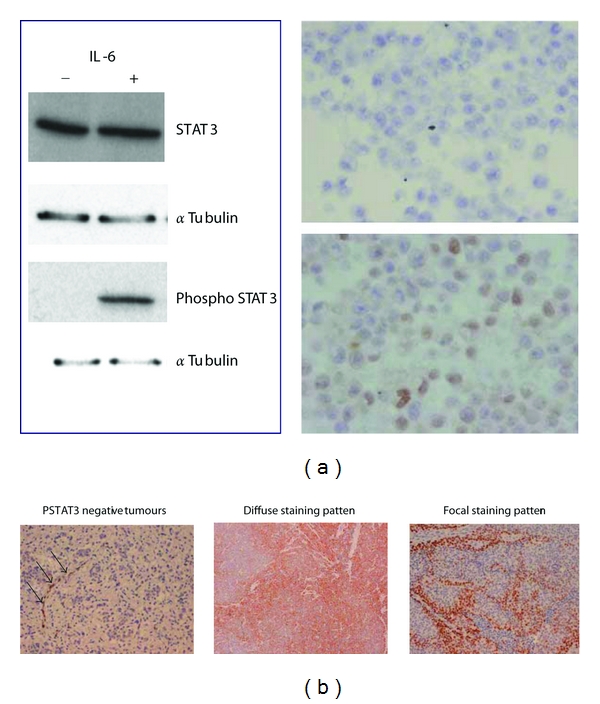
PSTAT3 staining identifies two distinct distribution patterns of PSTAT3 tumour cells in ESFT specimens. (a) Hela cells were cultured in the presence or absence of IL-6 prior to formation of cell pellets for generating lysate or embedding in paraffin following formalin fixation. A phospho-STAT3-specific antibody was used to probe lysates by Western blot or to stain cells by immunohistochemistry, magnification ×200. (b) Representative staining patterns of primary ESFT samples. In the left panel, tumor cells are P-STAT3 negative whereas endothelial cells stained positive for PSTAT3 (acting as an internal positive control, black arrows), magnification ×200.

Previous work has described a high incidence of STAT3 activation in ESFT [12], and we were interested in confirming this finding in an independent tissue set, as well as analysing tissue distribution of activated STAT3 within the tumor microenvironment. As a marker of STAT3 activation, we made use of a monoclonal antibody with specificity for the tyrosine^705^-phosphorylated form of STAT3 (P-STAT3) and first validated its specificity using IL-6-treated or -untreated HeLa cells (Figure 1(a)). We next stained a total of 31 ESFT primary tumors and found that 18 cores (58%) contained P-STAT3 positive tumour cells. Amongst these positive samples we identified 2 distinct distribution patterns of P-STAT3 staining (Figure 1(b)). Positive cells were either scattered throughout the tumor with no recognisable pattern (termed diffuse staining *n* = 11; 35%), or they existed in positively staining clusters (termed focal staining *n* = 7; 23%), particularly along fibrovascular bundles. The extent of P-STAT3 positive tumor cells amongst diffusely positive tumors was graded as follows: 8 tumors 1+; 2 tumors 2+; 1 tumor 3+.

### 3.2. Phosphorylated STAT3 Is Present in Ewing's Cell Lines, But Not PC3, Can Be Diminished by a STAT3 Inhibitor, and Reduces Viability in ESFT Cell Lines

**Figure 2:**
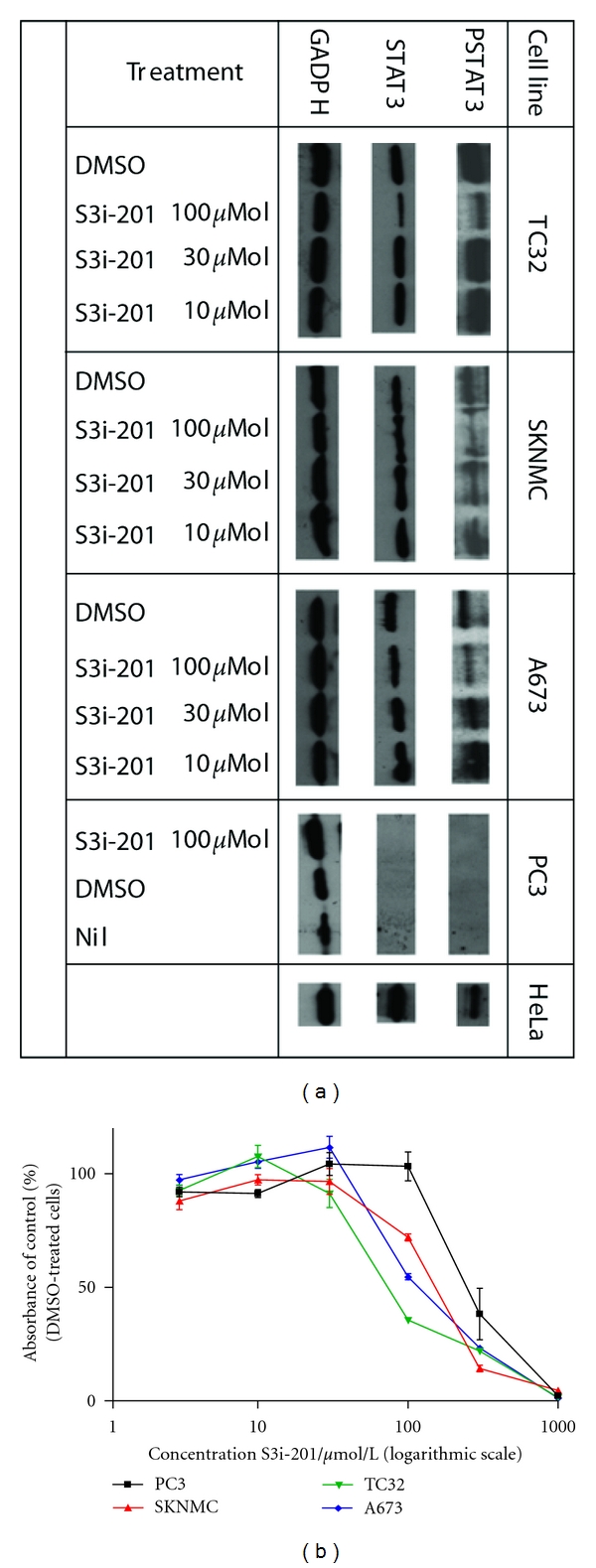
Phosphorylated STAT3 is present in Ewing's cell lines, but not PC3, which can be diminished by S3i-201 and reduces viability in ESFT cell lines. (a) Western blot analysis; Lysates from IL-6-stimulated and -unstimulated HeLa cells served as positive controls for P-STAT3 and total STAT3, respectively. The GADPH band is representative of loading in each lane. Data are representative of at least three independent experiments per cell line. (b) Cell viability is determined by MTT assay. Data are representative of at least three independent experiments per cell line.

We assessed the role of P-STAT3 in contributing to tumour growth by performing *in vitro* cell viability growth assays in a panel of ESFT cell lines and in the STAT3-null prostate cancer cell line, PC3. The presence or absence of P-STAT3 in the cell lines was demonstrated by Western blotting as was the ability of the specific STAT3 inhibitor, S3i-201 [14], to abolish phosphorylation of STAT3 (Figure 2(a)). Representative results of independent cell growth assays (at least three per cell line) are shown in Figure 2(b). Pharmacological inhibition of P-STAT3 by S3i-201 for 24 hours resulted in reduced tumour proliferation at a concentration of 100 *μ*mol/L in ESFT cell lines, but not in the STAT3-negative PC3 cell line. S3i-201 treatment nonspecifically inhibited viability in growth conditions in all cell lines (including the STAT3 negative PC3 cell line) at concentrations of 300 *μ*mol/L and 1000 *μ*mol/L, respectively.

### 3.3. STAT3 Inhibition in ESFT Cells Alters the Levels of a Limited Number of Chemokines

**Figure 3:**
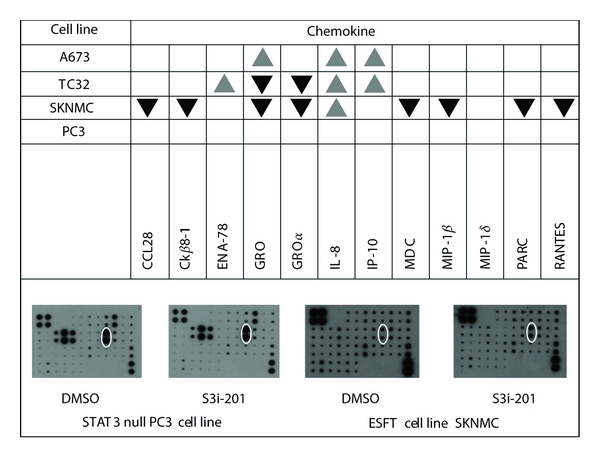
STAT3 inhibition in ESFT cells alters the levels of a limited number of chemokines. Upper panel the gray up-pointing triangle denotes an increase, and the black down-pointing triangle denotes a decrease, of more than 10%, observed in two independent experiments for each cell line. The changes represent a ratio of density values, comparing arrays of STAT3-inihibited cells with arrays of DMSO-treated cells. Lower panel examples of arrays performed on supernatants of STAT3 null control cells (PC3) and the ESFT cell line SKNMC, cultured in the absence (DMSO treatment) or presence (S3i-201 treatment) of STAT3 inhibition. C*κβ*8-1 = CCL23, ENA-78 = CXCL5, GRO = pan marker for GRO family chemokines, GRO*α* = CXCL1, IL-8 = CXCL8, IP-10 = CXCL10, MDC = CCL22, MIP-1*β* = CCL4, MIP-1*δ* = CCL15, PARC = CCL18, and RANTES = CCL5.

We next studied the effects of STAT3 inhibition on chemokine secretion patterns of ESFT cell lines and of the STAT3-negative PC3 cell line. Chemokine arrays were performed on supernatants obtained from cells cultured in the presence or absence of pharmacological P-STAT3 inhibition. For each cell line, two independent experiments were performed. Prior to analysis by chemokine arrays, we confirmed by Western blotting that P-STAT3 had been blocked in the cells from which the supernatants had been harvested (data not shown). Known STAT3-regulated chemokines (e.g., IL-8 [15]) were affected by P-STAT3 inhibition in all ESFT cell lines, but not in the STAT3-negative PC3 cell line. In addition to this, each ESFT cell line exhibited a distinct pattern of changes in chemokine levels shown in Figure 3.

## 4. Discussion

We sought to confirm that STAT3 is present in a subset of ESFT tumours and to investigate the role of STAT3 in ESFT. We were particularly interested in the role of STAT3 in contributing to tumour growth and immune regulation, which are classical roles described for STAT3 in adult tumours.

In our series, we demonstrated P-STAT3 positive tumour cells in 18/31 ESFT cores. In comparison, Lai et al. previously found P-STAT3 positive tumour cells in 25 out of 49 tumour cores [4]. A particular strength and distinguishing feature of our tissue arrays is the relatively large surface area of individual tissue cores (4 mm diameter), which allows us to assess tumour architecture. We made the observation that P-STAT3 positive cells were distributed in two distinct patterns, which we termed diffuse and focal. Although we are unable to comment on the significance of this observation, the level of organisation seen in the focal distribution pattern is intriguing. Given that around half of ESFT samples contained activated STAT3 tumour cells, we continued to investigate the role of STAT3 in ESFT *in vitro*.

We studied whether STAT3 contributes to tumour growth by performing viability assays of cells in growth conditions in a panel of three ESFT cell lines, which harbour constitutively activated STAT3. As a STAT3 negative control cell line, we used in our experiments the prostate cancer line PC3, which is homozygous STAT3 null [16]. As a pharmacological inhibitor of STAT3, we used S3i-201, which has been developed through structure-based virtual screening and targets the STAT3 SH2-domain [14]. We found that at 100 *μ*mol/L (previously reported IC50 of 86 ± 33 *μ*mol/L) S31-201 reduced viability of STAT3 positive ESFT tumour cells, but not of the STAT3 null PC3 cell line. At higher drug concentrations, cell viability was also diminished in the PC3 cell line, indicating STAT3-indepedent effects of S3i-201 at such concentrations. Our findings are consistent with data presented in the original drug discovery paper of S3i-201. Here Siddiquee et al. reported that in breast cancer cell lines S3i-201 inhibited tumour growth, with an IC50 of 86 ± 33 *μ*mol/L and with STAT3-independent cytotoxic effects at higher drug concentrations [14]. Our observations therefore lend support to the hypothesis that STAT3 plays a nonredundant role in contributing to growth of ESFT cell lines. The high IC50 of S3i-201 almost certainly disqualifies it from being a clinically usable drug.

Next we studied the effects of STAT3 inhibition on chemokine secretion patterns of ESFT cell lines through chemokine arrays performed on supernatants of ESFT cells. We confirmed STAT3 inhibition in the cells that produced the supernatants through Western blotting, and we used a STAT3 null cell line PC3 to exclude nonspecific effects. We found changes in chemokine levels of known STAT3 targets such as IL-8 whose secretion was increased by STAT3 inhibition in all three ESFT cell lines, but not in PC3. In addition, STAT3 inhibition induced a distinct the pattern of changes in chemokine secretion in each ESFT cell line. The significance of these findings is twofold. First, they suggest that STAT3 plays a role in regulating the chemokine environment of ESFT cell lines. Second, our observations highlight that in each ESFT cell lines STAT3 may be integrated into different immunological pathways.

## 5. Conclusion

In this study we confirm the finding that a subset of ESFT contains P-STAT3 positive tumour cells. Furthermore, we describe two novel distribution patterns of PSTAT3 positive tumour cells. Our *in vitro* experiments provide evidence that, in ESFT cell lines, STAT3 plays its classical role of contributing to tumour growth and regulating the tumour immune environment. As global efforts are underway to develop clinically usable STAT3 inhibitors, our findings have to be validated in different *in vitro* and *in vivo* models of ESFT, to provide a rationale for targeting STAT3 in patients suffering from ESFT.
